# ﻿Hidden pandemic: COVID-19-related stress, *SLC6A4* methylation, and infants’ temperament at 3 months

**DOI:** 10.1038/s41598-021-95053-z

**Published:** 2021-08-02

**Authors:** Livio Provenzi, Fabiana Mambretti, Marco Villa, Serena Grumi, Andrea Citterio, Emanuela Bertazzoli, Giacomo Biasucci, Lidia Decembrino, Rossana Falcone, Barbara Gardella, Maria Roberta Longo, Renata Nacinovich, Camilla Pisoni, Federico Prefumo, Simona Orcesi, Barbara Scelsa, Roberto Giorda, Renato Borgatti

**Affiliations:** 1 IRCCS Mondino Foundation, Pavia, Italy; 2 Scientific Institute IRCCS E. Medea, Bosisio Parini, Italy; 3 ASST Lodi, Lodi, Italy; 4 Guglielmo da Saliceto Hospital, Piacenza, Italy; 5 ASST Pavia, Pavia, Italy; 6 Fondazione IRCCS Policlinico San Matteo, Pavia, Italy; 7 University of Pavia, Pavia, Italy; 8 Università Bicocca, Milano, Italy; 9 San Gerardo Hospital, ASST Monza, Monza, Italy; 10 ASST Spedali Civili, Brescia, Italy; 11 University of Brescia, Brescia, Italy; 12 ASST Sacco Fatebenefratelli, Milan, Italy

**Keywords:** DNA methylation, Human behaviour, Paediatrics

## Abstract

The COVID-19 pandemic represents a collective trauma that may have enduring stress effects during sensitive periods, such as pregnancy. Prenatal stress may result in epigenetic signatures of stress-related genes (e.g., the serotonin transporter gene, *SLC6A4*) that may in turn influence infants’ behavioral development. In April 2020, we launched a longitudinal cohort study to assess the behavioral and epigenetic vestiges of COVID-19-related prenatal stress exposure in mothers and infants. COVID-19-related prenatal stress was retrospectively assessed at birth. *SLC6A4* methylation was assessed in thirteen CpG sites in mothers and infants’ buccal cells. Infants’ temperament was assessed at 3-month-age. Complete data were available from 108 mother-infant dyads. Greater COVID-19-related prenatal stress was significantly associated with higher infants’ *SLC6A4* methylation in seven CpG sites. *SLC6A4* methylation at these sites predicted infants’ temperament at 3 months.

## Introduction

Northern Italy was hit dramatically by the first COVID-19 wave during the first months of 2020^1^. With the exponential increase in the number of patients requiring intensive care and the mortality rate associated with this clinical condition, the COVID-19 pandemic is an unprecedented healthcare emergency that also emerges as a potential collective trauma^2,3^. The detrimental effects of COVID-19-related stress have been highlighted for healthcare professionals at the forefront of the emergency^4^ as well as for the general population^5^. Fragile individuals and those who are exposed to the pandemic during a period of heightened neuroplasticity may be especially at risk for the consequences of COVID-19-related stress^6–8^. Pregnancy is such a sensitive period for the embedding of environmental exposures—for bad and for good—in the developmental phenotype of infants^9^. Previous research suggested that the prenatal exposure to maternal stress may impact different domains of infant behavioral, affective, and socio-cognitive development^10–12^. Maternal stress during pregnancy may be specifically linked with alterations of the temperamental profile of the infant^13–15^. Previous studies reported that higher levels of prenatal stress were significantly associated with infants’ higher negative affectivity, lower expression of intense pleasure and difficulties in the regulatory functioning during the first months of life^15,16^. As temperament is involved in setting thresholds of reactivity to environmental stimuli^17^, understanding how adverse prenatal conditions may affect temperamental traits is crucial.

The influence of prenatal stress on later infants’ developmental outcomes may be at least partially mediated by epigenetic mechanisms^18^. DNA methylation is by far the most studied mechanism at the interface between environmental exposures and phenotype in human infants. This epigenetic mechanism mainly occurs among DNA sites rich in cytosine and guanine (i.e., CpG sites), it is involved in the regulation of genes’ expression and may contribute to gene silencing in a way that is highly susceptible to environmental stimuli^19,20^. DNA methylation is of specific concern when it occurs at the level of stress-related genomic portions, such as the serotonin transporter gene (*SLC6A4*)^21^. Previous research reported heightened CpG-specific *SLC6A4* methylation in infants exposed to prenatal maternal depression^22^ and stress as well as to postnatal adverse exposurs^23^. A recent systematic review reported that heightened *SLC6A4* methylation may be considered as a potential biomarker of early adverse experiences^21^. The study of the epigenetic vestiges of collective trauma has been useful to elucidate biomarkers of stress in previous large-scale events such as the Canadian Ice Storm in 1999^24^ or the World Trade Center terroristic attack in 2001^25^. Nonetheless, we do not know how a global pandemic may be harmful during pregnancy by increasing the exposure to stress and producing indirect behavioral effects on infants’ temperament. As such, in April 2020 we launched the Measuring the Outcomes of Maternal COVID-19-related Prenatal Exposure—i.e., MOM-COPE^26^—research project to assess the epigenetic and behavioral consequences of COVID-19-related prenatal stress on maternal well-being and infants’ development from birth to 12 months. In the present paper, we report on (1) the effects of COVID-19-related prenatal stress on maternal and infants’ *SLC6A4* methylation and (2) the association between infants’ *SLC6A4* methylation and temperament assessed at 3 months of age.

## Methods

### Participants and procedures

**Figure 1:**
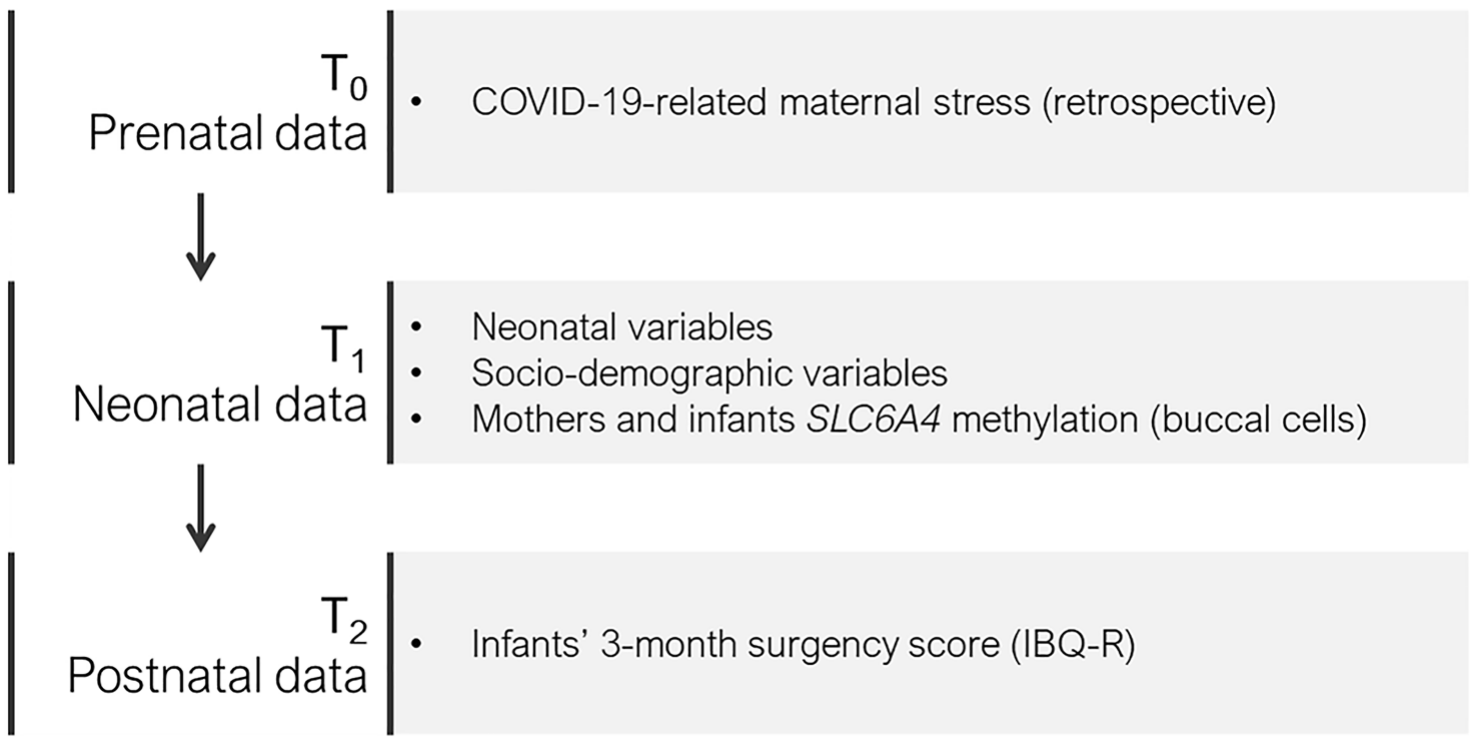
Overview of the study.

The MOM-COPE is a longitudinal and multi-centric cohort study that involves ten neonatal units in Northern Italy. The fully detailed description of this project is reported elsewhere^26^. Here we report on a sample of 108 (62%) mother-infant dyads who provided complete data for prenatal (T_0_), neonatal (T_1_) and 3-month (T_2_) assessments by February 2021 (Fig. 1). Mothers were included if at least 18-year-old, in the absence of prenatal and perinatal diseases or injuries, if they delivered at term (i.e., from 37 + 0 to 41 + 6 weeks of gestation), and if they were negative for COVID-19 at delivery. Mothers were first contacted at antepartum classes or immediately following the postpartum period. Socio-demographic and neonatal data were obtained from medical records. Within 48 h from delivery, the mothers filled in a first set of questionnaires to provide retrospective quantitative measures of prenatal COVID-19-related stress. Between 6 and 24 h, buccal cells were obtained from mothers and infants using DNA Genotek Oragene OC-175, according to manufacturer guidelines. When infants were approaching 3-month-age, mothers filled-in a questionnaire on infants’ temperament. The study was approved by the Ethics Committees of the project lead institution (IRCCS Mondino Foundation, Pavia, Italy) and the participating hospitals. All the procedures were performed in accordance with the 2018 Declaration of Helsinki for studies conducted with human participants. All mothers provided informed consent to participate to the study.

### Measures

Mothers self-reported on socio-demographic (i.e., age, educational level, occupational status). At delivery mothers retrospectively reported on their prenatal COVID-19-related stress during the last trimester of pregnancy through an ad-hoc questionnaire (Supplementary File S1). A mean COVID-19-related prenatal stress score was obtained, ranging from 1 (low) to 5 (high). Neonatal characteristics (i.e., gestational age, birth weight, head circumference, neonatal length, Apgar at minute 1, breastfeeding at birth, and mode of delivery) were collected from medical records. The saliva was collected from both mothers and infants using the OraCollect for Pediatrics kit OC-175 (DNA Genotek, Ottawa, Canada) between 6 and 24 h from delivery. Methylation assessment was conducted according to previous validated procedures from this lab^27,28^. The genomic DNA was extracted following manufacturer’s protocols and its quality was assessed using a Qubit fluorometer Invitrogen, Thermo Fisher Scientific, Waltham, Massachusetts, USA). The methylation status of the SLC6A4 gene’s region (chr17:28562750–28562958, 13 CpGs) was assessed by PCR amplification of bisulfite-treated DNA followed by Next Generation Sequencing (NGS) on a NEXTSeq-500 (Illumina, San Diego, California, USA). At infants’ 3-month-age, infants’ temperament was assessed with the short form version of the Infant Behaviour Questionnaire-Revised, IBQ-R^29^. The IBQ-R includes 91 items that are rated on a 7-point and summarized into 14 scales and 3 main factors: surgency (i.e., activity level and expression of pleasure), negative affectivity (i.e., distress, sadness and fear) and regulatory capacity (i.e., cuddliness, soothability and orienting).

### Plan of analysis

Descriptive statistics were computed for socio-demographic characteristics and for all the variables reported in the “Measures” section. Parametric indexes (i.e., mean, standard deviation, and range) were obtained for continuous variables, whereas frequencies and percentages were obtained for categorical variables. Normal distribution was checked for all variables of interest and normalization occurred by means of natural log (ln) adjustment. Bivariate Pearson’s correlation coefficients were computed to assess the association among COVID-19-related prenatal stress and both infants and mothers’ methylation status of *SLC6A4* CpG sites. Multiple-comparison bias was checked using the Benjamini–Hochberg procedure, *q* < 0.10. A principal component analysis (PCA) was used to reduce the number of methylation items; a single principal component (PC1) accounted for 35% of the total variance in *SLC6A4* methylation and it was used in further analyses. Bivariate correlations were carried to test for the potential association of neonatal and socio-demographic variables with both *SLC6A4* PC1 methylation and infants’ surgency at 3 months. A linear general model was used to estimate the association between COVID-19-related prenatal stress and infants *SLC6A4* PC1 methylation (ln). A linear general model was used to estimate the association between infants *SLC6A4* PC1 methylation and 3-month surgency score. Analyses were carried using R and IBM SPSS 26 for Windows, with *p* < 0.05.

## Results

**Figure 2:**
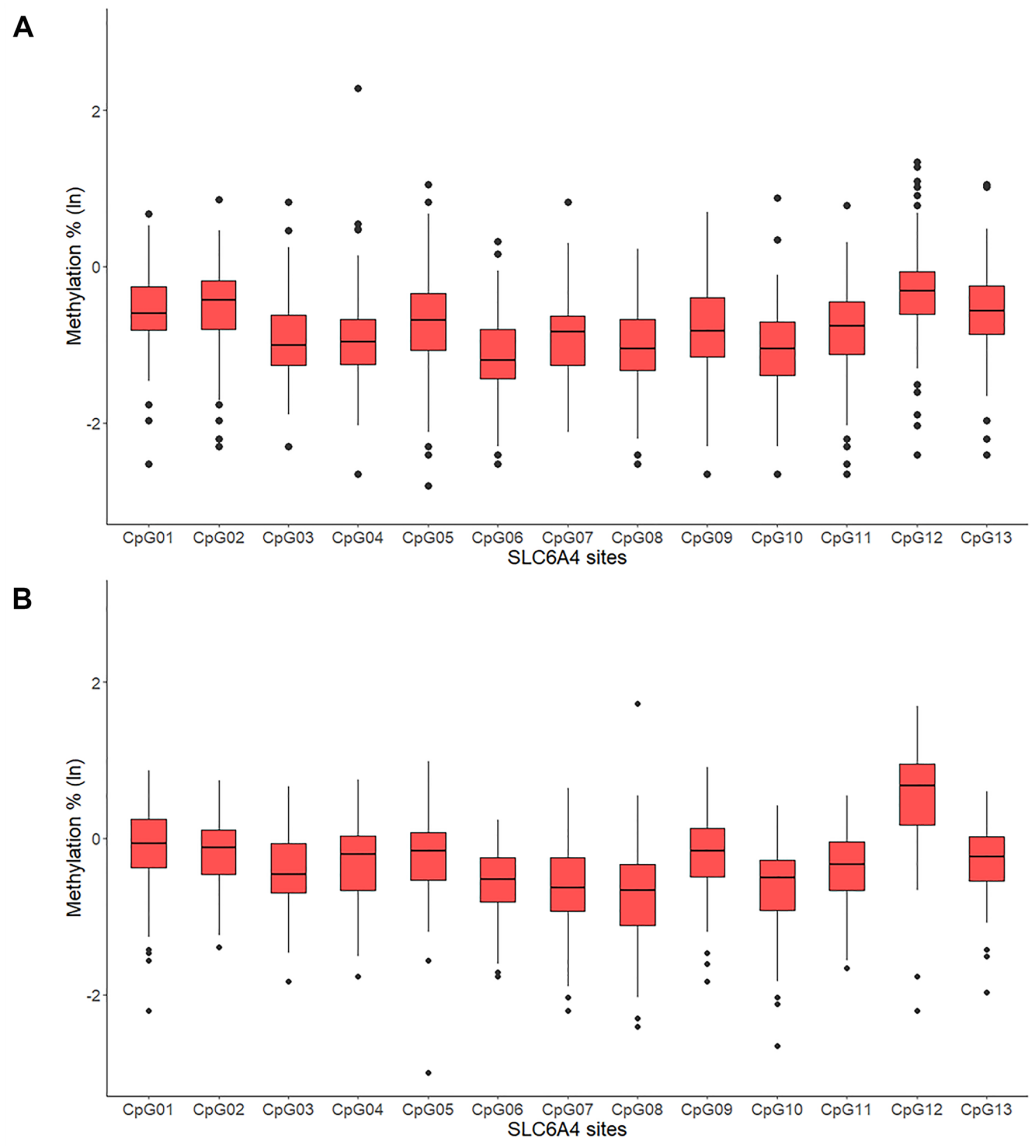
*SLC6A4* methylation in infants (**A**) and mothers (**B**). Note. Bars represent standard errors.

Descriptive statistics are reported in Table 1. Figure 2 reports infants and mothers’ CpG-specific *SLC6A4* methylation estimates.

**Table 1:** Descriptive statistics.

	Min	Max	Mean	SD
Gestational age (weeks)	37.00	42.00	39.71	1.05
Birth weight (g)	2430.00	4345.00	3342.88	413.82
Head circumference (cm)	30.00	39.00	34.33	1.27
Neonatal length (cm)	46.00	56.00	50.64	1.95
Apgar at minute 1	6.00	10.00	9.18	0.69
Maternal educational level (years of study)	5.00	23.00	14.44	3.57
Infants’ surgency	2.37	6.31	4.07	0.89
Infants’ negative affectivity	1.30	5.65	3.10	0.81
Infants’ regulatory capacity	3.52	6.81	5.15	0.76

			N	%
Maternal occupational status (employed)			95	88.0
Delivery (eutocic)			69	63.9
Infant's sex (females)			55	50.9
Breastfeeding at birth (maternal milk)			71	65.7

**Figure 3:**
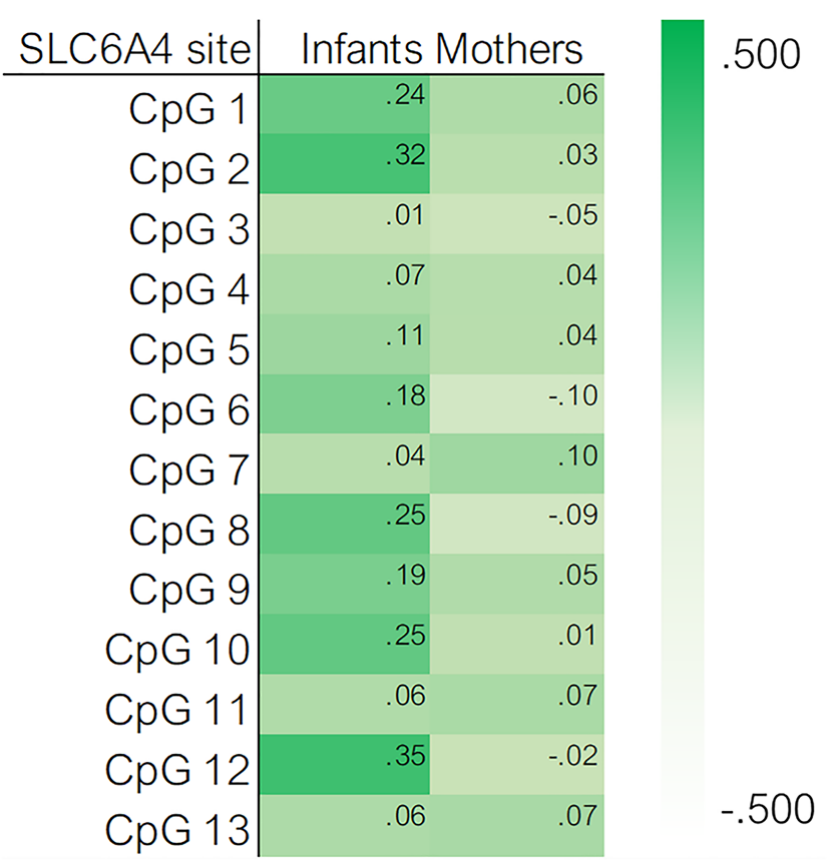
Heat map of the bivariate correlations between COVID-19-related prenatal stress and both infants and mothers’ *SLC6A4* methylation.

Bivariate correlations between COVID-19-related prenatal stress and both infants and mothers’ CpG-specific *SLC6A4* methylation are reported in Fig. 3. For infants, *SLC6A4* methylation was positively and significantly associated with COVID-19-related prenatal stress at seven CpG sites (i.e., 1, 2, 6, 8, 9, 10, and 12). All significant correlations survived to Benjamini–Hochberg test. No significant correlations emerged for mothers’ *SLC6A4* methylation.

The PCA results are reported in Table 2. The infants’ CpG sites whose methylation levels were significantly correlated with prenatal stress loaded on PC1, accounting for 35% of total variance in infants’ *SLC6A4* methylation.

**Table 2:** Principal component analysis (PCA) on infants’ *SLC6A4* methylation conducted among the 13 CpG sites.

SLC6A4 site	Principal components		
	PC1	PC2	PC3
CpG 1	**0.704**		
CpG 2	**0.735**		
CpG 3	0.512	**0.653**	
CpG 4			
CpG 5		**0.614**	
CpG 6	**0.691**		
CpG 7		**0.577**	
CpG 8	**0.635**		
CpG 9	**0.776**		
CpG 10	**0.618**		
CpG 11			**0.502**
CpG 12	**0.849**		
CpG 13			**0.793**

Loadings on the respective principal components (PC) are reported in bold. Loadings < 0.500 are not reported.

**Figure 4:**
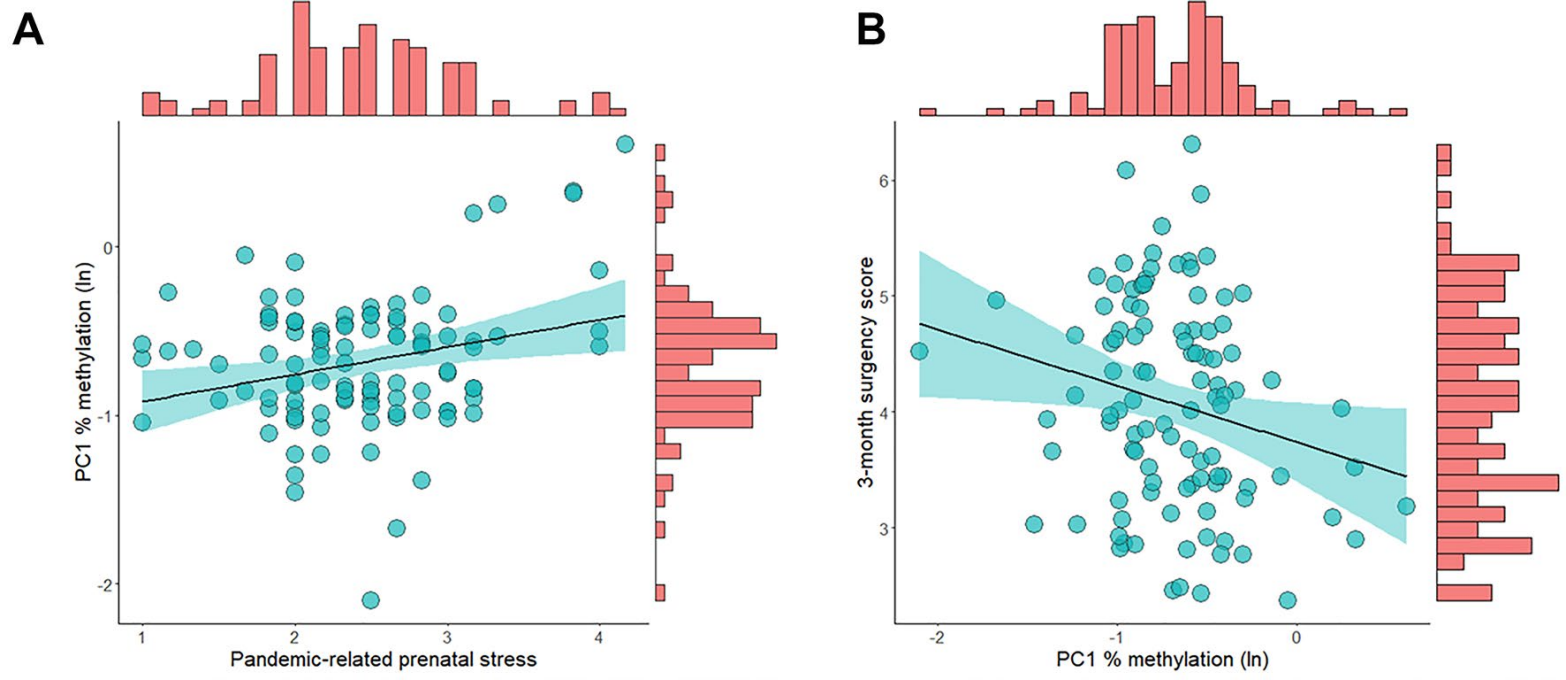
Association between (**A**) COVID-19-related prenatal stress and infants’ *SLC6A4* PC1 methylation; (**B**) infants’ *SLC6A4* PC1 methylation and 3-month surgency score.

PC1 showed non-Gaussian distribution and it was normalized by means of ln transformation. No significant correlations emerged for neonatal and socio-demographic variables with heightened PC1% methylation (ln) and infants’ 3-month temperament. COVID-19-related prenatal stress was significantly and positively associated with infants’ PC1% methylation (ln), *R*^2^ = 0.07, *F* = 7.71, *p* = 0.007, *B* = 0.16 [95% CI 0.05:0.29] (Fig. 4A). Infants’ PC1% methylation (ln) was significantly and associated with 3-month surgency score, *R*^2^ = 0.05, *F* = 5.05, *p* = 0.027, *B* = − 0.45 [95% CI − 0.92:− 0.06] (Fig. 4B): infants who showed higher levels of *SLC6A4* gene methylation in the selected region were rated by their parents as exhibiting greater surgency. No significant associations emerged for infants’ negative affectivity and regulatory capacity.

## Discussion

Pregnant women experiencing prenatal stress during the COVID-19 pandemic may give birth to infants who present heightened *SLC6A4* methylation levels and early dysregulations in the temperament profile at 3 months. These findings are consistent with previous literature that highlighted how precocious stress-related epigenetic signatures may represent a biomarker of early adversity exposures^21^.

Notably, in the present study, a significant effect of COVID-19-related prenatal stress emerged for infants’ *SLC6A4* methylation, but not for mothers. This result may be interpreted in the light of the conflict perspective in fetal programming by maternal stress^30^. Literature about maternal prenatal stress showed that it may act not only as a risk factor on fetal development^31^, but it may also lead to a fetus adaptive response, increasing his organism’s defensive processes^32^. Additionally, prenatal stress may represent a scenario of mother-fetus conflictual exchanges. Genetic interests of parents and offspring are only partially overlapping and—during critical conditions—the maternal biology may manipulate the fetal environment in order to promote its own biological interest^30^. From this perspective, it should be highlighted that maternal prenatal stress has been found to increase the risk of inflammations which, in turn, may further exposure the fetal compartment to stress hormones. One can speculate that, during the COVID-19 pandemic, the maternal biological functioning may have opted for the activation of protective mechanisms—such as cortisol production to reduce stress-induced inflammatory processes—that may have resulted in a heightened exposure of the fetus to stress hormones^33^. Unfortunately, in the present cohort study we did not collect placental tissues and the assessment of biomarkers of stress-related inflammations are not available.

In our sample, the *SLC6A4* methylation was significantly and negatively associated with infants’ temperament, in particular with the infants’ positive affect at 3 months. In a recent study, Gartstein and colleagues^34^ showed that the *SLC6A4* methylation was associated with soothability at 3 months in infants prenatally exposed to antidepressant medication, while Montirosso and colleagues^35^ found a significant impact on preterm infants’ duration of orienting and approach to novelty. However, these two studies did not document similar effects for control groups of healthy age-matched counterparts. In the present study, we enrolled only healthy full-term infants and mothers with no documented psychiatric conditions. As such, it can be hypothesized that the epigenetic effect observed at the level of the *SLC6A4* gene may be ascribed to the prenatal exposure to pandemic-related stress. In other words, although the present study lacks a control group of non-exposed age-matched mother-infant dyads, there is indirect support that the stress due to being exposed to a global healthcare emergency during pregnancy may contribute to the epigenetic regulation of the serotonin transporter gene in young infants.

## Limitations

First, we originally planned to have a control group enrolment between April and December 2021; nonetheless, due to the ongoing nature of the COVID-19 emergency in Italy, it was not possible to enroll non-exposed age-matched controls and this research project qualifies as a longitudinal cohort study. Second, the unprecedented nature of this healthcare emergency required to develop ad-hoc instruments to detect pandemic-related prenatal stress. Consistently, in our study, the prenatal stress was retrospectively assessed using an ad-hoc self-report measure. Although this measure was developed according to previous literature^26^, it provides an estimate of maternal stress during pregnancy that can be only partially compared to previous studies that included standardized measures. Third, *SLC6A4* methylation was peripherally assessed in buccal cells. The association between peripheral *SLC6A4* methylation and actual serotonin transporter expression has received partial confirmation in animal model research and cross-tissue concordance remains an open question^20^. Recently, Booij and colleagues reported on a significant association between *SLC6A4* methylation in buccal tissue and brain response to negative emotionality stimuli^36^, suggesting that peripheral markers of serotonin epigenetic regulation should be considered the closest reliable source of functionally relevant DNA methylation in human subjects. Finally, a single-gene approach to the study of the epigenetic correlates of early stress exposure in infants was made possible here by the availability of a well-documented rationale for the *SLC6A4* gene^20^. Nonetheless, it should be highlighted that multiple loci in different genomic regions may be potential targets of environmentally driven epigenetic regulation and other genes—e.g., *NR3C1*^37^, *BDNF*^38^—should be also involved.

## Conclusion

In a general population of healthy mother-infant dyads, pandemic-related prenatal stress emerged as a risk factor for infants’ development. In more specific terms, pregnant women experiencing greater COVID-19-related prenatal stress may give birth to infants who present higher levels of *SLC6A4* methylation and temperament dysregulation at 3 months. In this scenario, the consequences of COVID-19-related stress experienced during pregnancy risk to result in a “hidden pandemic” that may affect the early developmental trajectories of infants, even in absence of severe medical risk conditions. Appropriate actions are needed from clinicians and policymakers to timely provide families with appropriate and efficient preventive strategies during and after the healthcare emergency.

## Supplementary Information

Supplementary Information.
