# Supplementary material for: ﻿Hidden pandemic: COVID-19-related stress, SLC6A4 methylation, and infants’ temperament at 3 months
Source: Sci Rep. 2021 Aug 2;11:15658. doi: 10.1038/s41598-021-95053-z (PMC8329206; doi:10.1038/s41598-021-95053-z)
Supplement: Supplementary file 1 — Supplementary Information. [file 41598_2021_95053_MOESM1_ESM.docx]

**Supplementary File S1.** COVID-19-related prenatal stress questionnaire.

|  | During pregnancy… |
| --- | --- |
| 1 | How much worried were you about the risk of COVID-19 infection? |
| 2 | How much did you feel that your pregnancy was at risk due to COVID-19 pandemic? |
| 3 | How much did you fear for your health? |
| 4 | How much did you fear for your baby's health? |
| 5 | How much did you feel that you were losing confidence in your health? |
| 6 | How much did you feel you had lost faith in medicine? |

Note. Each item was rated from 1 (low) to 5 (high).
